# Effects of Histamine H_3_ Receptor Antagonist/Inverse Agonist Pitolisant on Temporal Prediction, Spatial Switching, and Spontaneous Locomotion in Mice

**DOI:** 10.1002/npr2.70115

**Published:** 2026-04-01

**Authors:** Shohei Kaneko, Kazuko Hayashi, Kota Yamada, Koji Toda

**Affiliations:** ^1^ Department of Psychology Keio University Tokyo Japan; ^2^ Japan Society for the Promotion of Science Tokyo Japan; ^3^ Institute for Quantitative Biosciences University of Tokyo Tokyo Japan

## Abstract

Histamine receptors contribute to a wide range of brain functions, including arousal, motivation, attention, and memory, but their causal involvement in switching behavior remains insufficiently understood. Here, we examined the effects of systemic administration of pitolisant, a histamine H_3_ receptor antagonist/inverse agonist, on adaptation to environmental changes in male mice using two behavioral experiments: a head‐fixed Pavlovian spatial switching temporal conditioning task and a freely‐moving open‐field assay. In the switching task, mice trained without external sensory cues rapidly adjusted both anticipatory and consummatory licking following a switch in reward location, demonstrating robust switching of temporal prediction to a new reward location. Pitolisant administration did not alter anticipatory licking, consummatory licking, or switching performance, indicating that H_3_ receptor blockade does not modulate temporal prediction and switching behavior in this assay. In contrast, high‐dose pitolisant markedly reduced spontaneous locomotor activity in the open‐field and fecal output, suggesting dose‐dependent effects on motor and autonomic function. Together, these findings indicate that histamine H_3_ receptor signaling exerts selective behavioral influences, with minimal impact on temporal prediction and spatial switching behavior under the present task conditions but clear effects on physiological and motor domains. This work refines the understanding of histaminergic modulation in complex behavior and highlights important considerations for interpreting the cognitive and noncognitive consequences of H_3_ receptor‐targeting therapeutics.

## Abstract 2

The histamine H_3_ receptor antagonist/inverse agonist pitolisant did not affect temporal prediction and spatial switching.

## Graphical abstract

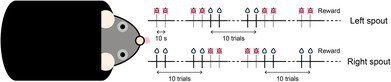

## 1 Introduction

Animals, including humans, inhabit environments that undergo continuous and often unpredictable change. Locations that were once safe may become dangerous, and previously reliable sources of food or shelter may disappear. Survival in such conditions depends critically on behavioral flexibility, which is the capacity to update previously learned associations when environmental contingencies shift. Reversal learning and rule‐switching tasks are widely used across species to assess this ability, as they repeatedly alter the task demands, requiring organisms to detect these changes and adapt their behavior accordingly [1]. Neurobiological studies have highlighted the orbitofrontal cortex, medial prefrontal cortex, striatum, and amygdala as essential nodes supporting such flexible updating, with serotonin, dopamine, and glutamate playing well‐established modulatory roles [1].

Although considerably less studied, the histaminergic system represents another neuromodulatory pathway that may contribute to behavioral flexibility. Histaminergic neurons originate in the tuberomammillary nucleus and project widely throughout the cortex, basal ganglia, hippocampus, and amygdala [2]—regions critically involved in reversal learning and rule‐switching tasks. Among the histamine receptor subtypes, the histamine H_3_ receptor has attracted particular interest due to its function as a presynaptic autoreceptor and heteroreceptor regulating the release of histamine and multiple other neurotransmitters, including dopamine, serotonin, and acetylcholine [3]. Importantly, H_3_ receptors are expressed in the prefrontal cortex, basal ganglia, and amygdala [4], positioning them as potential modulators of the very circuits implicated in flexible adaptation to environmental change.

Pharmacological studies further support this possibility. H_3_ receptor antagonist/inverse agonist increases acetylcholine, noradrenaline, and dopamine release [5], neuromodulatory actions known to influence cognitive switching and updating [1]. In rodents, H_3_ receptor antagonist/inverse agonist has been reported to improve attention, impulse control, and set‐shifting performance in some procedures [6], although results are inconsistent and appear to depend on task structure, drug dose, and species. These findings collectively suggest—but do not yet demonstrate—that H_3_ receptor signaling may contribute to adaptation to environmental changes measured via spatial switching tasks.

Pitolisant, a selective H_3_ receptor inverse agonist, is clinically approved for narcolepsy yet remains under‐characterized in basic behavioral neuroscience. Preclinical reports indicate that high doses can alter arousal, locomotion, autonomic output, and monoaminergic transmission [7], raising the possibility that systemic administration influences multiple behavioral domains beyond cognition. Thus, even when studying cognitive flexibility, it is critical to determine whether pitolisant alters general activity levels or autonomic arousal, both of which can confound performance in learning tasks. This consideration motivates the structure of the present study.

In Experiment 1, we examined whether pitolisant modulates spatial switching behavior in response to environmental changes in a head‐fixed Pavlovian switching task. Because H_3_ receptors regulate multiple neurotransmitter systems, we hypothesized that pharmacological enhancement of histaminergic tone might alter the ability to adapt to changing contingencies. In Experiment 2, we assessed the drug's effects on spontaneous locomotor activity, defecation, and urination in the open‐field test. These data were necessary for two reasons: (1) to rule out activity‐ or arousal‐related confounds that could masquerade as cognitive effects in Experiment 1, and (2) to provide foundational behavioral characterization of pitolisant in mice, addressing the current gap in preclinical literature. Although Experiment 2 does not test cognitive functions directly, its results are essential for interpreting performance in the switching task and for contextualizing pitolisant's broader physiological impact. Together, these experiments aim to clarify whether H_3_ receptor signaling contributes to adaptation to environmental change, and how pitolisant affects both specific cognitive processes and more general behavioral states.

## 2 Methods

### 2.1 Animals

Twenty adult male C57BL/6J mice were used in this study. All mice were experimentally naïve at the start of the procedures. Animals were maintained on a reversed 12‐h light–dark cycle (lights on at 20:00) under controlled temperature (24°C ± 2°C) and humidity (60% ± 20%). Experiments were conducted between 11:00 and 20:00, and each mouse was tested at approximately the same time of day throughout the study to minimize circadian variability. To reduce potential sources of bias, mice were randomly assigned to experimental conditions wherever applicable, and data collection and preprocessing were conducted by experimenters blinded to drug treatment. The sample size was determined based on previous studies using similar behavioral procedures and pharmacological manipulations. No animals were excluded from analysis. All procedures adhered to the ARRIVE guidelines. The protocols were approved by the Animal Care and Use Committee of Keio University.

### 2.2 Surgery

Mice were anesthetized with 1.0%–2.5% isoflurane mixed with room air and placed in a stereotaxic frame (942WOAE, David Kopf Instruments, Tujunga, CA, USA). A head post (H.E. Parmer Company, Nashville, TN, USA) was affixed to the skull using dental cement (Product #56849, 3M Company, Saint Paul, MN, USA) to allow head fixation during behavioral tasks. Prior to surgery, mice were group‐housed (two to four per cage). Following implantation, mice were housed singly during the recovery period and remained singly housed for the duration of training and testing to avoid confounding effects of social interactions. Postoperative monitoring was performed daily, and animals were allowed at least 1 week for recovery before training began. All surgical procedures included efforts to minimize pain and distress.

### 2.3 Behavioral Tasks

#### 2.3.1 Head‐Fixed Spatial Switching Temporal Conditioning Task

**FIGURE 1:**
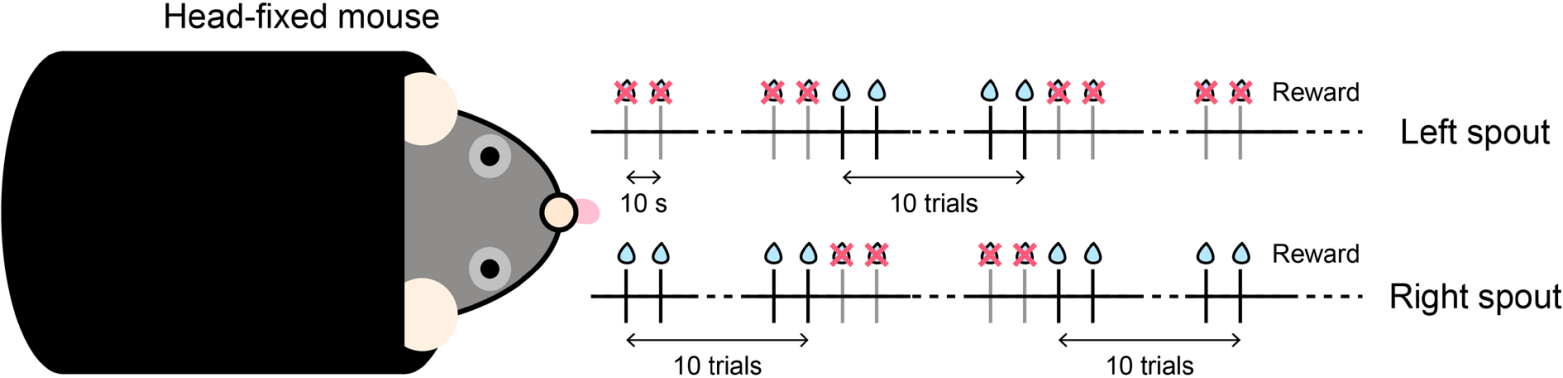
Head‐fixed spatial switching temporal conditioning task. Schematic illustration of the head‐fixed spatial switching temporal conditioning task.

To investigate whether the mice can learn to switch the spatial prediction of the reward, we trained the mice on a switching task [8] with no external sensory cues to signal the timing of the reward delivery and the switch of the rewarding location (Figure 1). Eight male mice ranging 4–6 months of age were used in the head‐fixed experiment. The mice were water‐deprived and received a 10% sucrose solution during the experiments. Their weights were monitored daily. In the head‐fixed experiment, we provided the 10% sucrose solution that is enough to maintain over 85% of free‐drinking weight of the mice. We also provided additional water after the experiment to maintain their weight as needed. The mice had unrestricted access to food in their cages. After recovery from surgery, the mice were water‐restricted in their home cage. On the first day of training, the mice were head‐fixed briefly and given water rewards to habituate them to the experimental environment. Behavioral experiments were conducted in a square behavioral chamber with a steel drinking spout placed directly in front of the animal's mouth. Each mouse was kept on a custom‐designed and 3D printed covered elevated platform, with its head fixed by two stabilized clamps holding the sidebars of the head post. The heights of the tunnel and clamps were aligned prior to each session to ensure comfort. The spout and meshed copper sheet under the stage were connected, and individual licking contacts between the mice and the drinking needle were recorded using a contact lickometer. Head‐fixed mice were allowed to lick the spout. For the initial training, we trained the fixed‐time schedule task with a single reward spout [8, 9, 10, 11]. Approximately 2 μL of 10% sucrose solution was delivered through the tube at 10 s intervals. Sucrose delivery and recording of licking data were conducted using custom‐made python3 (version 3.7.7) scripts with a custom‐made relay circuit with solenoids. On each day of the experiment, we ran one session that contained 250 trials. One trial consisted of a 10‐s interval and the reward delivery. We defined anticipatory licking as the number of licks from −5 to 0 s before the reward delivery and consummatory licking as the number of licks from 0 to +1 s after the reward delivery. After training on the fixed‐time schedule task, a switching task was initiated. The experimental setup of the switching task was identical to that of the fixed‐time schedule task, except that two drinking steel spouts were placed in front of the mouth of mice. The distance between each licking spout was set at 4.5 mm. Sucrose solution was delivered through one of the two spouts. The rewarding spout was switched every 10 trials. The amount of sucrose solution delivered was calibrated to be the same amount between each rewarding spout. Licking toward the rewarding spout was defined as the correct response, whereas licking toward the non‐rewarding spout was defined as an error response. We defined anticipatory licking as licking from 2 to 0 s before reward delivery, and consummatory licking as licking from 0 to 2 s after reward delivery. We defined the error rates as the number of lickings toward the non‐rewarding spout divided by the total number of lickings toward both spouts. The error rates in anticipatory licking are defined as the number of licking toward the non‐rewarding spout divided by the total number of licking toward both spouts within 2 s before the reward delivery. The error rates in consummatory licking are defined as the number of licking toward the non‐rewarding spout divided by the total number of licking toward both spouts within 2 s after the reward delivery. We defined the latencies of licking to the rewarding side of the spout by the timing from reward delivery to the first licking on that side.

#### 2.3.2 Open‐Field Task

To examine the effects of pitolisant on spontaneous locomotor activity, we conducted an open‐field task using twelve 2‐to 6‐month‐old adult male C57BL/6J mice. Habituation to the apparatus and saline injection were performed for 30 min per day for 3 days prior to the start of the experiment. The apparatus was a custom‐made white vinyl chloride box measuring 50 cm in length × 50 cm in width × 50 cm in height. Cameras (Logicool HD Webcam C922n, Logicool Co Ltd., Tokyo, Japan) were placed 106 cm above the bottom of the box. The video was recorded on a Windows PC. During habituation, the animals were placed in the open‐field immediately after receiving the intraperitoneal injection of saline and allowed to behave freely. The order of the saline and pitolisant 10 and 20 mg/kg injections was randomized. Because the half‐life of pitolisant is 10–12 h [5], we set 1–3 days of the interval between each session of the open‐field experiments to avoid the effect of pitolisant injection continuing until the next session. The duration of the experiment was 90 min. White noise (75 dB) was presented throughout experiment to mask the external noise. Every time after running the open‐field experiment, we wiped the inside of the box with 70%–80% alcohol and waited 30 min for drying.

### 2.4 Drug

We obtained pitolisant hydrochloride from Sigma‐Aldrich Co. LLC (St. Louis, MO, USA). Pitolisant was dissolved in a saline solution. We administered pitolisant to mice via intraperitoneal injection at a dose of 0.01 mL/g. Two conditions were used for pitolisant administration: high‐dose (20 mg/kg) and low‐dose (10 mg/kg). We determined the concentrations of low and high‐dose of pitolisant by referring to existing literature of experiments with mice [12, 13]. The concentrations of 10 and 20 mg/kg of pitolisant are within a range of commonly used dosage (approximately 0.2–20 mg/kg). In the head‐fixed temporal conditioning experiment (Experiment 1), the experiments were initiated 5 min after the injection of saline or pitolisant. In the open‐field experiment (Experiment 2), the experiments were initiated immediately after the injection of saline or pitolisant.

### 2.5 Analysis

RStudio (version 2022.02.0, RStudio PBC, MA, USA) and GraphPad Prism (version 9.3.1, GraphPad, CA, USA) were used for the analysis. In the open‐field experiments, we used bonsai to quantify locomotor activity in the open‐field box [14].

## 3 Results

### 3.1 Experiment 1: Spatial Switching Temporal Conditioning Task in Head‐Fixed Mice

To assess whether mice can learn to predict both the timing and location of a reward, we trained them on a spatial‐switching temporal conditioning task without external sensory cues. In this Pavlovian procedure, a 10% sucrose solution was delivered every 10 s via a blunt‐tipped needle positioned within the licking distance of head‐fixed mice, with the reward location switching every 10 trials. After training, all mice showed increased anticipatory licking at the correct timing and location, indicating spatio‐temporal prediction of reward delivery.

**FIGURE 2:**
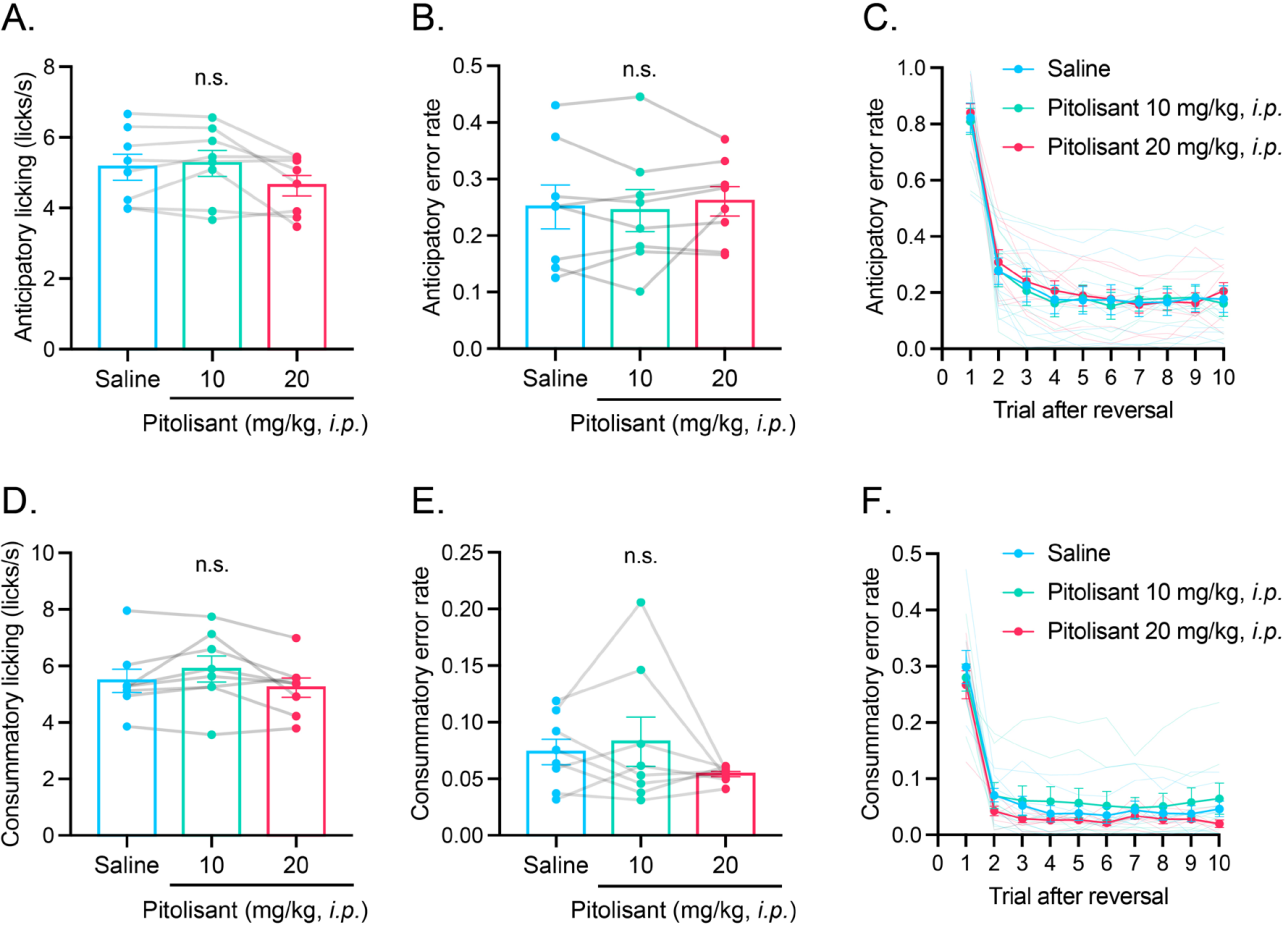
Effects of intraperitoneal injection of pitolisant on the performance in the head‐fixed spatial switching temporal conditioning task. (A) Effects of intraperitoneal injection of saline and pitolisant on anticipatory licking. (B) Overall anticipatory error rate following the injection of saline and pitolisant. (C) Anticipatory error rate after the switching of the rewarding location following the injection of saline and pitolisant. (D) Effects of intraperitoneal injection of saline and pitolisant on consummatory licking. (E) Overall consummatory error rate following the injection of saline and pitolisant. (F) Consummatory error rate after the switching of the rewarding location following the injection of saline and pitolisant. Error bars represent standard error of the mean. *N* = 8.

We next tested the role of histamine H_3_ receptors in this task by administering the antagonist/inverse agonist pitolisant intraperitoneally and measuring anticipatory and consummatory licking, as well as switching performance. Pitolisant produced almost no obvious effect on anticipatory licking (Figure 2A; repeated‐measures one‐way ANOVA: *F*(1.397, 9.777) = 4.749, *p* = 0.0458; all pairwise Tukey tests: *p* > 0.05) or consummatory licking (Figure 2D; repeated‐measures one‐way ANOVA: *F*(1.655, 11.59) = 3.792, *p* > 0.05). Similarly, error rates after switching were unaffected for both anticipatory (Figure 2B,C; repeated‐measures one‐way ANOVA: *F*(1.606, 11.24) = 0.3974, *p* > 0.05) and consummatory licking (Figure 2E,F; repeated‐measures one‐way ANOVA: *F*(1.289, 9.022) = 1.576, *p* > 0.05) under any pitolisant dose.

### 3.2 Experiment 2: Open‐Field Task in Free‐Moving Mice

**FIGURE 3:**
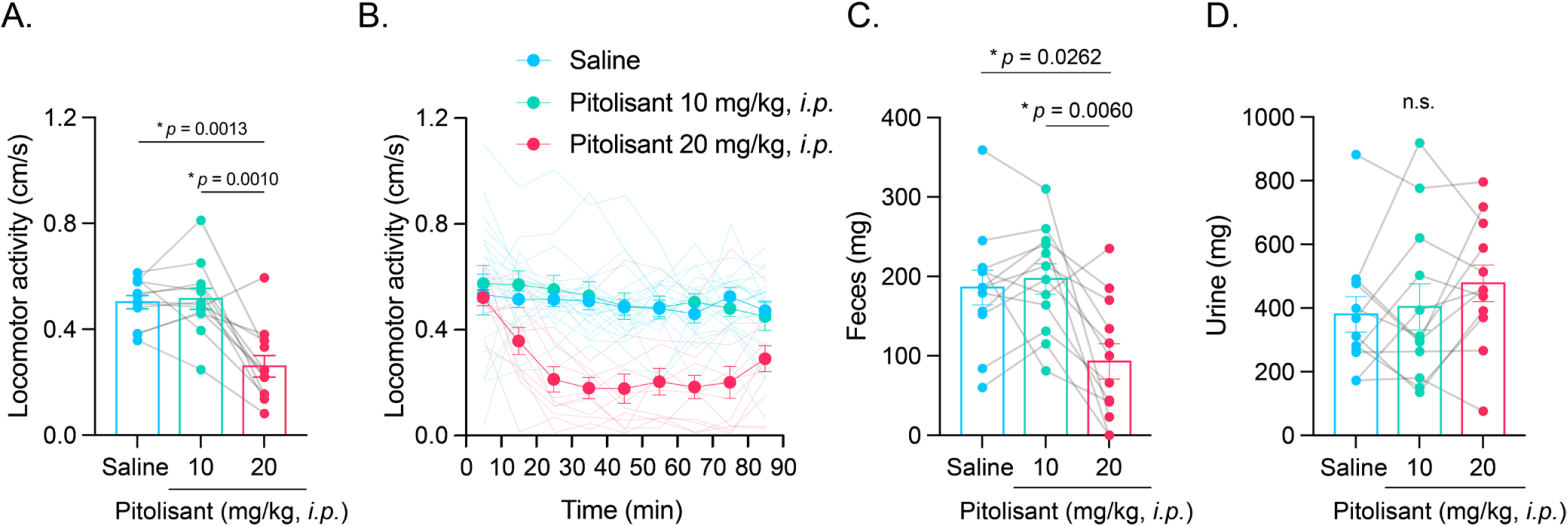
Effects of intraperitoneal injection of pitolisant on the performance in the freely‐moving open‐field task. (A) Overall locomotor activity in the open‐field task following saline or pitolisant injection. (B) Time course of open‐field activity after the start of the open‐field task following saline or pitolisant injection. (C) Fecal output collected after 90 min of the open‐field task. (D) Urine output collected after 90 min of the task. Error bars represent the standard error of the mean. *N* = 12.

To examine the effect of intraperitoneal injections of pitolisant on whole‐body motor function irrespective of licking movement, we examined the spontaneous locomotor activity of mice in the open‐field box. In this open‐field experiment, we also examined the effective durations of the pitolisant on the behavior, including the start and end time of the behavioral effect, regardless of the half‐life time of pitolisant. Thus, we started the open‐field experiment immediately after the injection of pitolisant. Spontaneous locomotor activity of the mice decreased in the high‐dose of pitolisant 20 mg/kg condition (Figure 3A; repeated measures one‐way ANOVA: *F*(1.551, 17.06) = 21.03, *p* < 0.0001; post hoc Tukey test: saline vs. pitolisant 20 mg/kg: *p* = 0.0013, pitolisant 10 mg/kg vs. pitolisant 20 mg/kg: *p* = 0.0010). Analysis of the locomotor activity in the time course during the open‐field task indicated that the effect of intraperitoneal injections of pitolisant was observed approximately 10–90 min after the injection (Figure 3B).

To assess the impact of pitolisant on autonomic nervous system function, we collected and quantified measured feces after the mice performed the open‐field task. Fecal output decreased in the high‐dose of pitolisant 20 mg/kg condition (Figure 3C; repeated measures one‐way ANOVA: *F*(1.480, 16.28) = 10.35, *p* = 0.0025; post hoc Tukey test: saline vs. pitolisant 20 mg/kg: *p* = 0.0262, pitolisant 10 mg/kg vs. pitolisant 20 mg/kg: *p* = 0.0060). There was no significant effect of pitolisant injection on the amount of urine (Figure 3D; repeated measures one‐way ANOVA: *F*(1.568, 17.24) = 1.880, *p* > 0.05).

## 4 Discussion

In this study, we examined the effects of intraperitoneal injection of pitolisant, a histamine H_3_ receptor antagonist/inverse agonist, on the performance of male mice in a head‐fixed Pavlovian spatial switching temporal conditioning task and in a freely‐moving open‐field task. In the switching task, mice were trained without any external sensory cues. After training, they rapidly reversed their anticipatory and consummatory licking in response to a change in the rewarding spout location, indicating the ability to switch spatial predictions. Pitolisant administration did not affect anticipatory and consummatory licking, temporal prediction, or response switching in the head‐fixed spatial switching temporal conditioning task. In addition, high‐dose pitolisant significantly reduced spontaneous locomotor activity in the open‐field task. The open‐field experiments also revealed that high‐dose pitolisant decreased fecal output, suggesting an effect on autonomic activity.

In Experiment 1, pitolisant did not significantly alter error rates in the spatial switching temporal conditioning task. This suggests that histamine H_3_ receptors may not play a critical role in switching between behavioral strategies, at least in the relatively simple version of the task used here, which imposed minimal mnemonic demands compared to procedures used in previous studies. Future work could employ more complex reversal learning procedures to further probe H_3_ receptor involvement in cognitive flexibility—for example, using two distinct auditory cues as conditioned stimuli and reversing their association with reward, thereby creating a contingency that cannot be solved by a simple win–stay/lose–shift strategy and would engage more demanding cognitive flexibility mechanisms.

In Experiment 2, high‐dose pitolisant (20 mg/kg) markedly reduced spontaneous locomotor activity. One possibility is that this behavioral effect arises from histaminergic projections to the striatum, a major component of the basal ganglia, which plays a central role in motor control and regulation. Pitolisant‐induced alterations in histaminergic modulation of striatal activity could underlie the observed suppression of locomotion. Another possibility is that pitolisant affects blood pressure or other physiological parameters. The robust locomotor suppression observed in the open‐field has implications for interpreting previous studies on pitolisant. For example, one study reported altered firing patterns in perirhinal cortex neurons following pitolisant administration in freely‐moving mice and proposed that histaminergic activity facilitates memory retrieval [13]. However, given the present evidence of reduced locomotion, decreased sensory input—particularly visual input—may also contribute to the observed changes in neuronal firing. Similarly, another study found prolonged freezing in a fear‐conditioning procedure after pitolisant administration and attributed it to enhanced memory retrieval [12]. Our data suggest that motor suppression should also be considered as an alternative explanation.

Although pitolisant reduced locomotor activity only after a delay of more than 20 min, suggesting that full engagement of H_3_ receptors may occur gradually, the absence of any drug‐related modulation across the entire switching session indicates that the null effect cannot be explained solely by the short interval between injection and task onset. If pitolisant had exerted even a delayed influence on cognitive processes, some deviation in performance would have been expected in the later phases of the task, but no such effects were observed. Nonetheless, future studies may benefit from initiating behavioral testing at least 20 min after administration to ensure complete receptor engagement.

A remaining limitation of the present study is that intraperitoneal administration of pitolisant does not allow us to determine which specific brain regions mediate its behavioral effects. Spontaneous locomotor activity and flexible, learning‐based behaviors are supported by partially distinct neural circuits—for example, basal ganglia pathways are essential for the regulation of locomotion, whereas prefrontal–striatal and hippocampal networks are critically involved in cognitive flexibility and reinforcement learning. Because systemic pitolisant engages the entire central histaminergic system, it remains unclear which projections or target regions contribute to the dissociable effects observed across the open‐field and switching tasks. Future studies employing region‐specific manipulations—such as local microinfusions, chemogenetic or optogenetic inhibition of histaminergic terminals, or conditional knockout approaches—will be necessary to identify the neural substrates through which H_3_ receptor blockade modulates spontaneous versus task‐dependent behaviors.

Another limitation of the present study is that we did not examine whether manipulations that increase central histamine levels—such as systemic administration of L‐histidine or inhibition of histamine N‐methyltransferase—would produce behavioral effects similar to or opposite to those observed with H_3_ receptor inverse agonism. Although these approaches enhance histaminergic transmission through mechanisms distinct from H_3_ receptor blockade, their physiological consequences are not necessarily equivalent. For example, elevating brain histamine content enhances tonic histaminergic tone but does not replicate the presynaptic disinhibition produced by H_3_ receptor inverse agonists, and downstream effects may differ across circuits that regulate locomotion and cognitive flexibility. Therefore, direct comparisons across these pharmacological strategies are not straightforward. Future work employing complementary manipulations of histamine synthesis, metabolism, and receptor function will be important for establishing whether the behavioral dissociations reported here reflect a specific role of H_3_ receptor signaling or a more general feature of histaminergic modulation.

The present findings should be interpreted in light of several methodological constraints inherent in our spatial switching temporal conditioning procedure. The fixed 10‐trial structure and constant 10‐s reward interval may have reduced the task's cognitive demands, potentially enabling animals to rely on sequential learning or anticipation of scheduled switches rather than fully detecting environmental change. Such predictability could mask subtle drug effects on switching behavior. Nonetheless, the task still required animals to update their choice behavior repeatedly across hundreds of trials, and the consistent trial‐by‐trial modulation observed in the saline groups indicates that mice were indeed sensitive to shifts in reward contingencies. Thus, while the design may underestimate the contribution of H_3_ receptor signaling to more complex forms of behavioral flexibility, it remains informative about the drug's influence on rapid spout‐specific updating under predictable rules. Future work should incorporate variable reversal lengths and asymmetric temporal contingencies to more rigorously probe flexibility in unpredictable environments. A procedure in which animals first establish a stable prediction before undergoing a true contingency reversal would be particularly valuable for dissociating pitolisant's effects on acquisition, maintenance, and reversal of learned associations. Together, such methodological refinements will allow a more definitive assessment of how H_3_ receptor inhibition shapes adaptive behavior across distinct dimensions of cognitive flexibility.

## 5 Conclusion

In summary, intraperitoneal administration of the H_3_ receptor inverse agonist pitolisant in head‐fixed mice performing a spatial switching temporal conditioning task provided no evidence for a critical role of H_3_ receptors in switching licking between spouts during this spatial switching task. In contrast, the open‐field experiments in freely moving mice revealed clear effects of pitolisant on locomotor activity and autonomic function, as indexed by excretion measures. These findings provide novel evidence for a role of histamine H_3_ receptors in motor regulation and physiological state, contributing to a more comprehensive understanding of histaminergic function in the brain.

## Author Contributions

K.T. designed the experiments. S.K. collected the data from the head‐fixed switching experiment with the help of K.Y. and K.T. S.K. collected the data from the open‐field task experiment with the help of K.H. and K.T. S.K., K.Y., and K.T. analyzed the data. S.K. and K.T. wrote the manuscript. S.K. and K.T. created all figures. S.K., K.H., K.Y., and K.T. discussed the data and commented on the manuscript. K.T. revised the manuscript accordingly.

## Funding

This research was supported by JSPS KAKENHI 18KK0070 (K.T), 19H05316 (K.T), 19K03385 (K.T), 19H01769 (K.T), 20J21568 (K.Y), 22H01105 (K.T), 23H02787 (K.T), 23K27478 (K.T), 23K22376 (K.T), 24H00729 (K.T), 24K16869 (K.Y), 24KJ0069 (K.Y), 24K06626 (K.H), and 25KJ0306 (K.H). Keio Gijuku Fukuzawa Memorial Fund (K.T), Keio Academic Development Fund (K.T), Smoking Research Foundation (K.T), and HOKUTO Foundation for the Promotion of Biological Science (K.T).

## Ethics Statement

The experimental and housing protocols were approved by the Animal Care and Use Committee of Keio University.

## Consent

The authors have nothing to report.

## Conflicts of Interest

The authors declare no conflicts of interest.

## Supporting information


**Data S1:** npr270115‐sup‐0001‐DataS1.xlsx.

## Data Availability

The data that support the findings of this study are available in the Supporting Information of this article.
